# Comparison of 6-Month and Prolonged Dual Antiplatelet Therapy after Percutaneous Coronary Intervention with Biodegradable Polymer Everolimus-Eluting Stent

**DOI:** 10.1155/2022/2914385

**Published:** 2022-09-28

**Authors:** Yong-Hoon Yoon, Gyung-Min Park, Jae-Hyung Roh, Sung-Ho Her, Seong-Hoon Lim, Tae Soo Kang, Seung Jin Lee, Jang-Whan Bae, WoongGil Choi, Yong-Mo Yang, Junghee Kim, Yu Jeong Choi, Si Wan Choi, Jae-Hwan Lee

**Affiliations:** ^1^Department of Cardiology, Chungnam National University Sejong Hospital, Chungnam National University College of Medicine, Sejong, Republic of Korea; ^2^Department of Cardiology, Ulsan University Hospital, University of Ulsan College of Medicine, Ulsan, Republic of Korea; ^3^Department of Cardiology, Saint Vincent's Hospital, College of Medicine, The Catholic University of Korea, Suwon, Republic of Korea; ^4^Division of Cardiovascular Medicine, Department of Internal Medicine, Dankook University Hospital, Dankook University College of Medicine, Cheonan, Republic of Korea; ^5^Department of Cardiology, Soonchunhyang University Cheonan Hospital, Soonchunhyang University College of Medicine, Cheonan, Republic of Korea; ^6^Department of Internal Medicine, Chungbuk National University Hospital, Chungbuk National University College of Medicine, Cheongju, Republic of Korea; ^7^Division of Cardiology, Department of Internal Medicine, Konkuk University College of Medicine, Chungju, Republic of Korea; ^8^Division of Cardiology, Department of Internal Medicine, Cheongju St. Mary's Hospital, Cheongju, Republic of Korea; ^9^Department of Cardiology, Daejeon Sun Medical Center, Daejeon, Republic of Korea; ^10^Division of Cardiology, Eulji University Hospital, Eulji University School of Medicine, Daejeon, Republic of Korea; ^11^Department of Cardiology, Chungnam National University Hospital, Chungnam National University College of Medicine, Daejeon, Republic of Korea

## Abstract

**Background:**

The optimal duration of dual antiplatelet therapy (DAPT) after biodegradable-polymer (BP) everolimus-eluting stent (EES) implantation remains uncertain.

**Methods:**

This study analyzed 793 patients who underwent percutaneous coronary intervention (PCI) with BP-EES in 10 cardiovascular centers in Korea between July 2016 and January 2018. Using the prescription data at 6 months post-PCI, we divided these patients into two groups, namely, short-DAPT and prolonged-DAPT groups, which underwent DAPT for 6 and > 6 months of PCI, respectively. The primary endpoint, which included mortality, myocardial infarction, or target-vessel revascularization at 2 years, was compared by propensity score (PS) matching between the two groups.

**Results:**

Out of the 793 patients, 283 matched pairs were identified by PS matching. Out of this matched population, 405 (71.6%) patients had an acute coronary syndrome. The primary endpoint did not differ in 2 years between the short-DAPT and prolonged-DAPT groups (7.5% vs. 8.3%; hazard ratio, 0.87; 95% confidential interval, 0.47–1.60; *P* = 0.648). Likewise, no difference was found regarding mortality, cardiac mortality, myocardial infarction, target-lesion failure, target-vessel failure, and bleeding events defined by the Bleeding Academic Research Consortium and Thrombolysis In the Myocardial Infarction classification. Meanwhile, one patient in the short-DAPT group had definite stent thrombosis at 364 days post-PCI. Subgroup analysis showed that several anatomical and procedural factors were not significantly related to DAPT duration. Most patients (77.4%) in both groups were prescribed clopidogrel at discharge.

**Conclusions:**

In real-world patients undergoing PCI with BP-EES, the ischemic and bleeding endpoints demonstrated no difference between 6-month and prolonged (>6 months) DAPT.

## 1. Introduction

The optimal duration of dual antiplatelet therapy (DAPT) after percutaneous coronary intervention (PCI) remains controversial. In the current US and European guidelines, the recommended duration of DAPT is ≤6 months for stable coronary artery disease and ≤12 months for acute coronary syndrome (ACS) after PCI with a drug-eluting stent (DES) [1–3]. Nonetheless, the rate of stent thrombosis has been remarkably reduced through the development of a newer-generation drug-eluting stent with a thin-strut, leading to shorter DAPT trials, especially in high-bleeding risk populations [4–7]. Recent several landmark randomized trials evaluated the impact of a shorter duration of DAPT compared with that of the conventional duration (usually 1 year) on ischemic clinical outcomes as well as bleeding events [8–12]. The results were promising; in fact, the shorter DAPT duration gradually replaces the currently recommended longer DAPT duration in clinical practice.

The polymer, which is a coating material on the stent's metallic surface, carries the needed amount of antiproliferative drugs for a specific time [13]. It enables the constant release of drugs to inhibit neointima proliferation inside coronary stents and reduces the rate of in-stent restenosis compared with the bare-metal stent [13, 14]. However, the persistence of durable polymers induces arterial healing impairment that can cause fatal complications, such as stent thrombosis [15]. Furthermore, polymer-induced chronic inflammation triggers neoatherosclerosis, which progresses to stent failure at longer periods, thereby requiring additional interventions [15]. To overcome polymer-induced adverse effects after PCI with DES, biodegradablepolymer (BP) was developed, which disappears within several months after implantation [16, 17]. Thus, various types of stents with BP have been introduced in clinical practice. However, the optimal duration of DAPT after BP everolimus-eluting stent (EES) implantation remains unclear. Therefore, we assessed the efficacy and safety of short-term (6 months) DAPT and compared it with prolonged DAPT (>6 months) in real-world patients who underwent PCI with BP-EES.

## 2. Methods

### 2.1. Study Design and Population

The DC-Synergy registry is a prospective all-comer real-world registry that has been recording patients over 18 years of age who underwent PCI with BP-EES in 10 cardiovascular centers in Korea since July 2016. Patients registered between July 2016 and January 2018 were enrolled in this study. BP-EES (SYNERGY™, Boston Scientific, US), which was used in this study, was characterized by a thin platinum-chromium platform (74–81 *μ*m) with BP (PDLLA, 4 *μ*mol/L) at the outer surface of the scaffold for everolimus (100 *μ*g/cm^2^) release. [16, 17] BP was designed to be released entirely at 4 months. The exclusion criteria were as follows: (1) experience of PCI combined with other types of coronary stent, (2) life expectancy <1 year, and (3) cardiogenic shock or severe pulmonary edema before the index procedure. We also excluded patients with no baseline or clinical follow-up data, no prescription of any antiplatelet at 6 months after PCI, and no prescription of oral anticoagulants at baseline. The institutional review boards of the participating centers approved the research protocol, and all eligible patients provided written informed consent.

### 2.2. Study Procedure, Data Collection, and Clinical Follow-up

Treatment strategies that include decision-making in terms of PCI, access route, devices to be used for the procedure, and the number, length, and diameter of the stent were at the discretion of the attending interventional cardiologists according to each patient's demographic and angiographic factors. DAPT was initiated one day before the elective PCI or just before the emergent PCI. Medical treatment after the index procedure was guided by current guidelines and established standards of practice. After the index PCI, the patients were followed up at 1 month, 3 months, 6 months, 1 year, and annually thereafter. DAPT was prescribed for at least 6 months, and its prolonged use or switch to single antiplatelet therapy (SAPT) was at the discretion of the attending physician. Any single antiplatelet agent such as aspirin (100–325 mg once daily) or P2Y_12_ inhibitors (e.g., 75 mg of clopidogrel once daily, 90 mg of ticagrelor twice daily, or 10 mg of prasugrel once daily) can be administered beyond 6 months after PCI. Information such as patient demographics, combined comorbidity, angiographic characteristics, laboratory findings, and follow-up outcomes was obtained from the medical records acquired during regular visits or telephone interviews. Data were collected in a web-based, dedicated electronic case-reporting form and periodically monitored by research personnel.

### 2.3. Definition and Study Endpoints

Short-DAPT was defined as the use of DAPT for 6 months after PCI. The duration was based on the prescription data at 6 month follow-up. If there was a prescription switch from DAPT to SAPT at 6 months follow-up, those were categorized as the short-DAPT group, whereas those who continued DAPT >6 months were categorized as the prolonged-DAPT group.

The primary endpoint of this study was the composite of all-cause mortality, myocardial infarction (MI), or target-vessel revascularization (TVR) at 2 years after the index procedure. The secondary ischemic endpoints of interest included composites of cardiac mortality, MI, or TVR; all-cause mortality or MI; and cardiac mortality or MI; and all-cause mortality, cardiac mortality, MI, TVR, target-lesion revascularization (TLR), and definite or probable stent thrombosis at 2 years. Cardiac mortality was defined as any death caused by a cardiovascular problem or if there was no identified cause of death. MI was defined as cardiac enzyme increase beyond the upper reference limit, with ischemic symptoms or signs that occurred spontaneously during the follow-up. Periprocedural MI was not accounted for the event in this study. TVR was defined as any revascularization procedure of the coronary vessels that were the target of the index procedure, and TLR was defined as the repeat revascularization within a 5 mm border of the index procedure stent. Bleeding events were recorded according to the Bleeding Academic Research Consortium (BARC) and Thrombolysis in Myocardial Infarction (TIMI) classification [18, 19]. For the outcome analysis, we assessed the BARC grades 2, 3, or 5, and TIMI major or minor bleeding. All clinical outcomes were confirmed by the source documentation obtained from each hospital, and an independent group of clinicians conducted central adjudication for all clinical events. The PRECISE-DAPT score was calculated using a web-based calculator to assess the bleeding risk in the study population (https://precisedaptscore.com/).

### 2.4. Statistical Analysis

Patient demographics and procedural characteristics are presented as means with standard deviations in continuous variables and as numbers with percentages in categorical variables. Comparisons between groups were performed using the Pearson's chi-square test for the categorical variables and the student's *t*-test for the continuous variables. To compensate for the non-randomized design of this study, we performed 1 : 1 propensity score (PS) matching to adjust the selection bias of DAPT prescription at 6 months with a caliper of 0.2. The covariates that were input in the logistic regression model determining the PS were as follows: age, sex, initial presentation (stable angina, unstable angina, non--ST-segment elevation MI, or ST-segment elevation MI), atrial fibrillation, body mass index, hypertension, diabetes, diabetes on insulin, current smoker, dyslipidemia, previous MI, congestive heart failure, peripheral vascular disease, chronic kidney disease, chronic kidney disease on hemodialysis, left ventricular ejection fraction, target vessel, multivessel stent, stent number, mean stent diameter, stent length, access route, complete revascularization, and discharge medications (beta-blocker, calcium channel blocker, angiotensin-converting enzyme inhibitor or angiotensin-receptor blocker, and statin). To assess the appropriateness of the adjustment, we used standardized mean differences between both groups after PS matching. Survival curves and event rates in the matched population were estimated using the Kaplan-Meier method. The risk of short-DAPT on clinical outcomes of interest was investigated and compared with that of prolonged DAPT by using Cox proportional hazards models. The time-dependent effect of DAPT duration during the follow-up period was assessed by landmark analysis in the survival curve. All reported *P* values were two-sided, and *P* < 0.05 was considered statistically significant. All statistical data were analyzed using the R software (version 3.5.1, R Foundation for Statistical Computing, Vienna, Austria).

## 3. Results

### 3.1. Study Population, Demographics, and PS Matching

Of the 972 patients enrolled in the DC-Synergy registry between July 2016 and January 2018, 179 were excluded (Supplemental Figure 1). Finally, a total of 793 patients were included in this study. The mean age of the study population was 65.4 ± 10.9, and 580 (73.1%) were men. Acute coronary syndrome (ACS) comprised 65.8% of the total study population. Compared with the prolonged-DAPT group, the patients in the short-DAPT group tended to have a more stable presentation, higher body mass index, lower chronic kidney disease prevalence, higher left ventricular ejection fraction, lesser stents, lesser use of femoral approach, and a higher rate of complete revascularization rate than the prolonged-DAPT group (Table 1). There were no statistically significant differences in age, presence of diabetes, previous MI, target coronary vessels, stent size, and discharge medications between both groups.  Table 2 shows the matched study cohort based on PS matching, and the standardized mean differences were <0.1 for all variables, indicating that the short-DAPT and prolonged-DAPT groups were balanced well after adjustment. PRECISE-DAPT scores were 14.3 ± 8.3 and 15.2 ± 8.4 in short-DAPT and prolonged-DAPT groups in the total population (*P*=0.165), and 15.0 ± 8.7 and 15.1 ± 8.4 in short-DAPT and prolonged-DAPT groups in the matched population (*P*=0.965).

### 3.2. Clinical Outcomes

**Figure 1:**
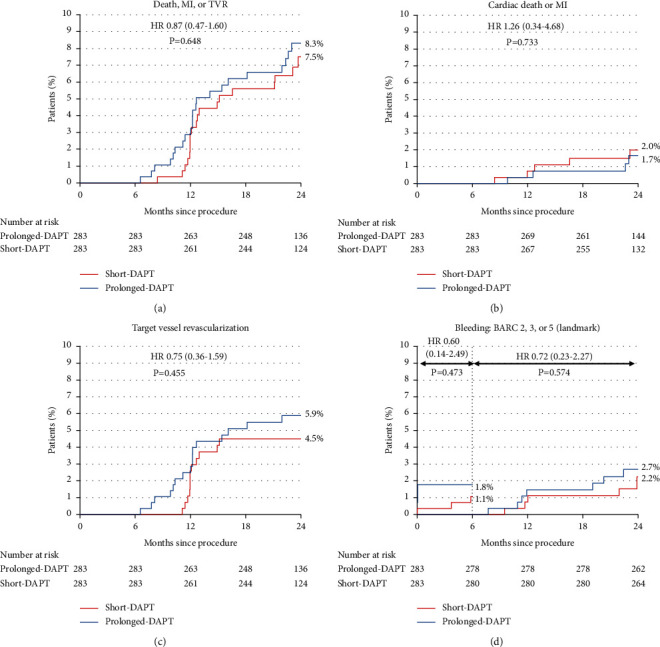
Kaplan-Meier survival curves for primary and secondary endpoints in the matched population. BARC, bleeding academic research consortium; DAPT, dual antiplatelet therapy; HR, hazard ratio; MI, myocardial infarction; TVR, target-vessel revascularization. (a) Death, MI, or TVR. (b) Cardiac death or MI. (c) Target vessel revascularization. (d) Bleeding: BARC 2, 3, or 5 (landmark).

For the primary endpoint, the composite of all-cause mortality, MI, or TVR occurred in 19 (7.5%) and 22 (8.3%) patients in the short-DAPT and prolonged-DAPT groups at 2 years (*P*=0.648) in the matched population (Table 3 and Figure 1). The rate of other composite endpoints, including cardiac mortality, MI, or TVR, and all-cause mortality or MI, also demonstrated no differences. Mortality occurred in 12 patients in both groups, with no differences in all-cause and cardiac mortality. TVR occurred in 12 (4.5%) and 16 (5.9%) patients in the short-DAPT and prolonged-DAPT groups, respectively, with no clinically significant difference (*P*=0.455). The TLR rates were similar in both groups. Only one patient from the short-DAPT group manifested definite stent thrombosis at 364 days after PCI and underwent repeated intervention for the treatment. Bleeding events (BARC 2, 3, or 5) occurred in 20 patients in the matched population within 2 years. At 2 years, the two groups demonstrated no differences (3.3% vs. 4.4%, *P*=0.650) during the entire follow-up period and after 6 months of PCI (2.2% vs. 2.7%, *P*=0.574) in the landmark analysis. Supplemental Table 1 and Supplemental Figure 2 show the clinical outcomes and Kaplan-Meier curves in the overall population before PS matching. The primary endpoint rate was significantly lower in the short-DAPT group (5.0% vs. 8.9%, *P*=0.030) than in the prolonged-DAPT group, which had a significantly higher rate of TVR (2.8% vs. 6.7%, *P*=0.012).

**Figure 2:**
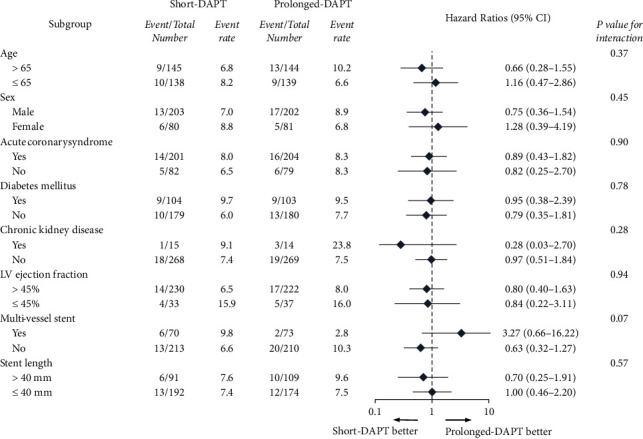
Subgroup analysis for the primary endpoint in the matched population. DAPT, dual antiplatelet therapy; LV, left ventricle.

Figure 2 illustrates the effect of short-DAPT on the primary outcome according to several subgroups, including the clinical and procedural factors of the matched population. The short-DAPT group in patients undergoing multivessel stenting tended to have worse adverse events. Nevertheless, initial ACS presentation, presence of diabetes, and stent length were not significantly related to poorer outcomes in the short-DAPT group.

In both groups, 77.4% of patients in the matched population were prescribed clopidogrel at discharge, whereas 22.6% were prescribed potent P2Y_12_ inhibitors (ticagrelor and prasugrel) (Supplemental Table 2). Potent P2Y_12_ inhibitors were switched to clopidogrel during the first 6 months in 10 patients in each group. At 6 months, aspirin was continually administered, while P2Y_12_ inhibitors were discontinued in 226 patients (79.9%) in the short-DAPT group.

## 4. Discussion

In this study involving a real-world population, we evaluated the clinical efficacy and safety of short-DAPT (6 months) in patients who underwent PCI with BP-EES. The major findings of the study are as follows: (1) compared with prolonged-DAPT, short-DAPT was related to similar efficacy in preventing ischemic cardiovascular events at 2 years after PCI; (2) short-DAPT was not related to reduced bleeding outcomes defined by BARC and TIMI classification; and (3) short- and prolonged-DAPT strategies had no significant interaction with several clinical subgroups.

The role of DAPT is to reduce the risk of ischemic events, especially stent thrombosis, during the early phase after PCI with DES. Considering those patients who have an increased risk of ischemic events, current guidelines recommend the use of DAPT for 12 months in patients with ACS and 6 months in patients with stable angina [1–3]. With the advances in technology, second-generation DES with a thin strut was developed over the last 2 decades, contributing to the lowered incidence of ischemic events after PCI [4–7]. However, prolonged use of DAPT is unavoidably associated with subsequent bleeding during long-term follow-up, especially in the high-bleeding risk population [20, 21]. Thus, many randomized clinical trials and observational studies have challenged the recommended use of DAPT for 6 or 12 months with a shorter duration of DAPT, and the results are promising.

Approximately 70% of our study patients had ACS at baseline. The results demonstrated similar rates of the ischemic composite endpoints between the short- and prolonged-DAPT groups after PCI with BP-EES. The ischemic and bleeding risks vary over time after PCI, and the ischemic risk is greater in the early phase following the ACS event. Thus, current guidelines for ACS recommend prolonged DAPT, which is twice as long as that for stable angina patients [3, 22]. Recently, several large randomized clinical trials with newer-generation stents tested 1–3 months of DAPT in patients with ACS; such duration was even shorter than that in our study. The TWILIGHT, SMART-CHOICE, and TICO trials showed that 3-month DAPT is not inferior in preventing cardiovascular events with lower bleeding rates compared with the 12-month DAPT in the stable angina or ACS population [8–10]. The STOPDAPT-2 trial tested the 1-month DAPT followed by clopidogrel monotherapy for up to 1 year; results showed that a composite of cardiovascular and bleeding events in the 1-month DAPT was superior in patients undergoing PCI with cobalt-chromium EES [12]. Meanwhile, the GLOBAL LEADERs trial showed no clinical benefit of ticagrelor monotherapy after 1 month of PCI [11]. However, data regarding the efficacy and safety of switching DAPT to a single antiplatelet agent at 6 months after BP-EES implantation in real-world patients remain limited. Most randomized trials used potent P2Y_12_ inhibitors, usually ticagrelor, for maintaining SAPT. Our study demonstrated that a shorter DAPT duration followed by SAPT (mostly aspirin) could be a safe option after PCI for patients with ACS (70%), consistent with the previous randomized trials.

Considering the varying risks of ischemia or bleeding events among patients, the duration of DAPT after PCI should be carefully selected for each patient. Current guidelines recommend a shorter duration of DAPT in patients with a high-bleeding risk in both the ACS and stable angina populations [2, 3]. In contrast, prolonged use for DAPT up to 1 year might be beneficial for some populations with a low bleeding risk, as presented in a meta-analysis that showed that prolonged use of DAPT is related to lower MI risk [23]. In our study, several clinical and procedural factors had no significant interactions with DAPT duration except for the tendency of higher ischemic risk in short-DAPT among patients who underwent multivessel stenting. Therefore, further studies are warranted to evaluate which persons can benefit from short or prolonged DAPT according to various clinical and anatomical factors.

### 4.1. Limitations

First, there is the inherent limitation of the nonrandomized design in this study. Although PS matching analysis was used to adjust for potential selection bias, unmeasured confounders that may have affected the results could not be excluded. In addition to the baseline characteristics, comorbidities associated with a worse prognosis were observed more frequently in the prolonged-DAPT group. Since the duration of DAPT use was determined by the attending physician, there may be a systematic bias across the study that low-risk patients were selectively treated with short-DAPT and high-risk patients were more frequently treated with prolonged-DAPT. Second, the initial design of this registry was not powered to calculate the differences in clinical outcomes between the short-and prolonged-DAPT groups. In addition, the lack of statistical differences in the clinical outcomes in both groups could be underestimated by the small sample size with low clinical event rates. Furthermore, bleeding events could not be evaluated because of the extremely low incidence during the study follow-up. Third, short and prolonged DAPT were categorized only by the prescription data at 6 months after PCI. Therefore, the compliance of the study patients in the administration of all antiplatelet agents could not be assessed in the study result. Fourth, different types and potency levels of antiplatelet agents were used during study follow-up because antiplatelet agents were selected according to the discretion of the attending cardiologists in the real-world clinical practice. According to the results of recent randomized trials, the use of potent P2Y_12_ inhibitors is favored when switching from DAPT to SAPT. Therefore, our results should be interpreted with caution because most SAPT was prescribed with aspirin, and potent P2Y_12_ inhibitors were used in a minimal number.

## 5. Conclusions

In this study, which compared the clinical outcomes of short-DAPT (6 months) and prolonged-DAPT (>6 months) in patients who underwent PCI with BP-EES, there were no differences in cardiovascular events rate during 2 years of follow-up. However, patients in the short-DAPT group undergoing multivessel stenting tended to have worse adverse events than the prolonged-DAPT group. The bleeding event was not different in both groups, but the event number and rate were extremely small.

## Figures and Tables

**Table 1:** Baseline characteristics in the total population.

	Short-DAPT (*N * = 483)	Prolonged-DAPT (*N * = 310)	Standardized mean difference	*P*
Age	65.1 ± 11.0	65.8 ± 10.7	0.070	0.341
Male	357 (73.9)	223 (71.9)	0.044	0.595

Initial presentation			0.241	0.012
Stable angina	167 (34.6)	83 (26.8)		
Unstable angina	201 (41.6)	143 (46.1)		
Non–ST-segment elevation MI	70 (14.5)	37 (11.9)		
ST-segment elevation MI	45 (9.3)	47 (15.2)		

Atrial fibrillation	19 (3.9)	5 (1.6)	0.142	0.099
Body mass index	24.9 ± 3.1	24.4 ± 3.2	0.159	0.029
Hypertension	313 (64.8)	191 (61.6)	0.066	0.404
Diabetes	156 (32.3)	117 (37.7)	0.114	0.134
Diabetes on insulin	15 (3.1)	17 (5.5)	0.117	0.140
Current smoker	130 (26.9)	80 (25.8)	0.025	0.793
Dyslipidemia	240 (49.7)	139 (44.8)	0.097	0.207
Previous MI	37 (7.7)	29 (9.4)	0.061	0.477
Congestive heart failure	8 (1.7)	3 (1.0)	0.060	0.619
Peripheral vascular disease	13 (2.7)	14 (4.5)	0.098	0.237
Chronic kidney disease	18 (3.7)	23 (7.4)	0.161	0.033
Chronic kidney disease on dialysis	8 (1.7)	17 (5.5)	0.207	0.005
LV ejection fraction	60.9 ± 10.8	58.4 ± 12.5	0.174	0.003

Target vessel				
(1) Left main	29 (6.0)	28 (9.0)	0.115	0.142
(2) Left anterior descending artery	279 (57.8)	186 (60.0)	0.045	0.582
(3) Left circumflex artery	140 (29.0)	90 (29.0)	0.001	>0.999
(4) Right coronary artery	160 (33.1)	122 (39.4)	0.130	0.087

Multivessel stent	104 (21.5)	83 (26.8)	0.123	0.107
Number of stents	1.5 ± 0.8	1.7 ± 0.9	0.205	0.005
Mean diameter of stent	3.2 ± 0.4	3.1 ± 0.4	0.115	0.117
Stent length	37.3 ± 25.4	41.6 ± 26.5	0.168	0.020
Overlapping stenting	65 (13.5)	47 (15.2)	0.026	0.570
Bifurcation stenting	5 (1.0)	6 (1.9)	0.065	0.455
Noncompliance balloon postdilatation	459 (95.0)	296 (95.5)	0.033	0.904
IVUS use	135 (28.0)	73 (23.5)	0.151	0.196
Femoral approach	140 (29.0)	114 (36.8)	0.166	0.027
Complete revascularization	315 (65.2)	175 (56.5)	0.180	0.016

Discharge medication				
(1) Beta-blocker	305 (63.1)	178 (57.4)	0.117	0.124
(2) Calcium channel blocker	124 (25.7)	86 (27.7)	0.047	0.574
(3) ACE inhibitor or ARB	283 (58.6)	200 (64.5)	0.122	0.111
(4) Statin	444 (91.9)	299 (96.5)	0.194	0.016

PRECISE-DAPT score	14.3 ± 8.3	15.2 ± 8.4	0.101	0.165

ACE, angiotensin-converting enzyme; ARB, angiotensin-receptor blocker; DAPT, dual antiplatelet therapy; LV, left ventricle; MI, myocardial infarction.

**Table 2:** Baseline characteristics of the matched population.

	Short-DAPT (*N* = 283)	Prolonged-DAPT (*N* = 283)	Standardized mean difference	*P*
Age	65.6 ± 11.0	66.0 ± 10.7	0.035	0.676
Male	203 (71.7)	202 (71.4)	0.008	>0.999

Initial presentation			0.046	0.960
Stable angina	82 (29.0)	79 (27.9)		
Unstable angina	133 (47.0)	131 (46.3)		
Non-ST-segment elevation MI	33 (11.7)	34 (12.0)		
ST-segment elevation MI	35 (12.4)	39 (13.8)		

Atrial fibrillation	6 (2.1)	5 (1.8)	0.026	>0.999
Body mass index	24.7 ± 3.1	24.5 ± 3.2	0.074	0.378
Hypertension	175 (61.8)	178 (62.9)	0.022	0.862
Diabetes	104 (36.7)	103 (36.4)	0.007	>0.999
Diabetes on insulin	13 (4.6)	14 (4.9)	0.017	>0.999
Current smoker	77 (27.2)	72 (25.4)	0.040	0.703
Dyslipidemia	132 (46.6)	133 (47.0)	0.007	>0.999
Previous MI	19 (6.7)	23 (8.1)	0.054	0.630
Congestive heart failure	1 (0.4)	3 (1.1)	0.084	0.616
Peripheral vascular disease	11 (3.9)	12 (4.2)	0.018	>0.999
Chronic kidney disease	15 (5.3)	14 (4.9)	0.016	>0.999
Chronic kidney disease on dialysis	8 (2.8)	8 (2.8)	<0.001	>0.999
LV ejection fraction	60.3 ± 11.1	59.4 ± 11.8	0.078	0.355

Target vessel				
(1) Left main	20 (7.1)	26 (9.2)	0.078	0.442
(2) Left anterior descending artery	173 (61.1)	172 (60.8)	0.007	>0.999
(3) Left circumflex artery	78 (27.6)	81 (28.6)	0.024	0.852
(4) Right coronary artery	103 (36.4)	103 (36.4)	<0.001	>0.999

Multivessel stent	70 (24.7)	73 (25.8)	0.024	0.847
Number of stents	1.6 ± 0.9	1.6 ± 0.9	0.041	0.627
Mean diameter of stent	3.1 ± 0.4	3.1 ± 0.4	0.035	0.674
Stent length	40.2 ± 26.7	40.9 ± 26.5	0.029	0.732
Femoral approach	89 (31.4)	95 (33.6)	0.045	0.654
Overlapping stenting	43 (15.2)	41 (14.5)	0.020	0.906
Bifurcation stenting	3 (1.1)	5 (1.8)	0.060	0.722
Noncompliance balloon postdilatation	268 (94.7)	269 (95.1)	0.016	>0.999
IVUS use	82 (29.0)	66 (23.3)	0.129	0.151
Complete revascularization	156 (55.1)	161 (56.9)	0.036	0.735

Discharge medication				
(1) Beta-blocker	166 (58.7)	163 (57.6)	0.021	0.865
(2) Calcium channel blocker	74 (26.1)	76 (26.9)	0.016	0.924
(3) ACE inhibitor or ARB	178 (62.9)	179 (63.3)	0.007	>0.999
(4) Statin	271 (95.8)	272 (96.1)	0.018	>0.999

PRECISE-DAPT score	15.0 ± 8.7	15.1 ± 8.4	0.004	0.965

ACE, angiotensin-converting enzyme; ARB, angiotensin-receptor blocker; DAPT, dual antiplatelet therapy; LV, left ventricle; MI, myocardial infarction.

**Table 3:** Primary and secondary endpoints between the short-DAPT and prolonged-DAPT groups in the matched population.

	Short-DAPT (*N * = 283)	Prolonged-DAPT (*N * = 283)	Hazard ratio	*P*
Primary endpoint				
Mortality, MI, or TVR	19 (7.5)	22 (8.3)	0.87 (0.47–1.60)	0.648

Secondary endpoints				
Cardiac mortality, MI, or TVR	16 (6.1)	19 (7.2)	0.84 (0.43–1.64)	0.615
Mortality or MI	8 (3.4)	7 (2.8)	1.16 (0.42–3.20)	0.775
Cardiac mortality or MI	5 (2.0)	4 (1.7)	1.26 (0.34–4.68)	0.733
Mortality	7 (3.0)	5 (2.0)	1.42 (0.45–4.48)	0.547
Cardiac mortality	4 (1.6)	2 (0.9)	2.01 (0.37–10.98)	0.420
MI	1 (0.4)	2 (0.8)	0.50 (0.05–5.54)	0.574
TLR	8 (3.0)	9 (3.3)	0.89 (0.34–2.30)	0.809
TVR	12 (4.5)	16 (5.9)	0.75 (0.36–1.59)	0.455
Stent thrombosis, definite or probable	1 (0.4)	—	—	—
Bleeding, BARC 2, 3, or 5	8 (3.3)	12 (4.4)	0.67 (0.27–1.63)	0.650
Bleeding, TIMI major or minor	7 (2.7)	9 (3.4)	0.80 (0.30–2.14)	0.376

BARC, bleeding academic research consortium; MI, myocardial infarction; TIMI, thrombolysis In myocardial Infarction; TLR, target-lesion revascularization; TVR, target-vessel revascularization.

## Data Availability

The data that support the findings of this study are available from the corresponding author upon reasonable request.

## Supplementary Materials

Supplementary Materials Supplemental Table 1: shows a higher rate of mortality, MI, or TVR in an unmatched population. Supplemental Table 2: shows the similar outcomes between short-DAPT and prolonged-DAPT groups after the inverse probability of treatment weighting. Supplemental Table 3: summarized the treads of antiplatelet uses in the study population. Supplemental Figure 1: shows the study flow. Supplemental Figure 2: reveals the Kaplan–Meier curves of unmatched population. Supplemental Table 1: primary and secondary endpoints between the short-DAPT and prolonged-DAPT groups in the total population. Supplemental Table 2: primary and secondary endpoints between the short-DAPT and prolonged-DAPT groups in the total population with inverse probability of treatment weighting. Supplemental Table 3: antiplatelet agents used during follow-up in the matched population. Supplemental Figure 1: Study Flow. Supplemental Figure 2: Kaplan–Meier survival curves for primary and secondary endpoints in the total population.
