# Supplementary material for: Comparison of 6-Month and Prolonged Dual Antiplatelet Therapy after Percutaneous Coronary Intervention with Biodegradable Polymer Everolimus-Eluting Stent
Source: Cardiol Res Pract. 2022 Sep 28;2022:2914385. doi: 10.1155/2022/2914385 (PMC9534716; doi:10.1155/2022/2914385)

**Supplementary Online Content**

**I. Supplemental Tables**

**Supplemental Table 1.** Primary and Secondary Endpoints between the Short-DAPT and Prolonged-DAPT Groups in the Total Population

**Supplemental Table 2.** Primary and Secondary Endpoints between the Short-DAPT and Prolonged-DAPT Groups in the Total Population with Inverse Probability of Treatment Weighting

**Supplemental Table 3.** Antiplatelet Agents Used during Follow-up in the Matched Population

**Supplemental Figure. 1.** Study Flow

**Supplemental Figure. 2.** Kaplan–Meier Survival Curves for Primary and Secondary Endpoints in the Total Population

**Supplemental Table 1.**

|  | Short DAPT | Prolonged DAPT | Hazard ratio | P |
| --- | --- | --- | --- | --- |
| Mortality, MI, or TVR | 22 (5.0) | 26 (8.9) | 0.53 (0.30–0.94) | 0.030 |
| Cardiac mortality, MI, or TVR | 17 (3.8) | 23 (7.9) | 0.46 (0.25–0.87) | 0.017 |
| Mortality or MI | 10 (2.4) | 7 (2.6) | 0.93 (0.35–2.44) | 0.878 |
| Cardiac mortality or MI | 5 (1.2) | 4 (1.5) | 0.81 (0.22–3.00) | 0.747 |
| Mortality | 9 (2.2) | 5 (1.8) | 1.17 (0.39–3.50) | 0.777 |
| Cardiac mortality | 4 (0.9) | 2 (0.8) | 1.29 (0.24–7.04) | 0.769 |
| MI | 1 (0.2) | 2 (0.7) | 0.32 (0.03–3.55) | 0.355 |
| TLR | 9 (1.9) | 12 (4.0) | 0.47 (0.20–1.13) | 0.091 |
| TVR | 13 (2.8) | 20 (6.7) | 0.41 (0.20–0.82) | 0.012 |
| Stent thrombosis, definite or probable | 1 (0.2) | 0 | - | - |
| Bleeding, BARC 2, 3 or 5 | 9 (2.1) | 14 (4.7) | 0.41 (0.18–0.95) | 0.037 |
| Bleeding, TIMI major or minor | 8 (1.8) | 10 (3.4) | 0.52 (0.20–1.31) | 0.166 |

BARC, Bleeding Academic Research Consortium; MI, myocardial infarction; TIMI, Thrombolysis In Myocardial Infarction; TLR, target-lesion revascularization; TVR, target-vessel revascularization

**Supplemental Table 2.**

|  | | | Short DAPT | Prolonged DAPT | | Hazard ratio | P | |  |
| --- | --- | --- | --- | --- | --- | --- | --- | --- | --- |
|  | Death, MI, or TVR | 23 (5.4) | | | 22 (7.6) | 0.67 (0.37-1.22) | | 0.194 | |
|  |  |  | | |  |  | |  | |
|  | Cardiac death, MI, or TVR | 18 (4.0) | | | 19 (6.8) | 0.59 (0.30-1.13) | | 0.109 | |
|  | Death or MI | 10 (2.6) | | | 6 (2.1) | 1.21 (0.45-3.25) | | 0.703 | |
|  | Cardiac death or MI | 5 (1.2) | | | 3 (1.2) | 1.04 (0.27-3.97) | | 0.954 | |
|  | Death | 10 (2.4) | | | 4 (1.5) | 1.57 (0.52-4.81) | | 0.426 | |
|  | Cardiac death | 4 (1.1) | | | 2 (0.6) | 1.76 (0.31-10.00) | | 0.526 | |
|  | MI | 1 (0.2) | | | 2 (0.6) | 0.33 (0.03-3.65) | | 0.367 | |
|  | TLR | 9 (2.0) | | | 11 (3.6) | 0.55 (0.22-1.38) | | 0.206 | |
|  | TVR | 14 (3.0) | | | 17 (5.8) | 0.51 (0.24-1.05) | | 0.068 | |
|  | Stent thrombosis, definite or probable | 1 (0.2) | | | 0 | - | | - | |
|  | Bleeding, BARC 2, 3 or 5 | 12 (2.8) | | | 13 (4.3) | 0.59 (0.24-1.43) | | 0.243 | |
|  | Bleeding, TIMI major or minor | 11 (2.3) | | | 10 (3.5) | 0.68 (0.25-1.81) | | 0.438 | |

BARC, Bleeding Academic Research Consortium; MI, myocardial infarction; TIMI, Thrombolysis In Myocardial Infarction; TLR, target-lesion revascularization; TVR, target-vessel revascularization

**Supplemental Table 3.**

|  | Aspirin | | Clopidogrel | | Ticagrelor | | Prasugrel | |
| --- | --- | --- | --- | --- | --- | --- | --- | --- |
| Prescription | Short DAPT | Prolonged DAPT | Short DAPT | Prolonged DAPT | Short DAPT | Prolonged DAPT | Short DAPT | Prolonged DAPT |
| At discharge | 283 (100) | 283 (100) | 219 (77.4) | 219 (77.4) | 48 (17.0) | 57 (20.1) | 16 (5.7) | 7 (2.5) |
| At 1 month | 283 (100) | 283 (100) | 226 (79.9) | 229 (80.9) | 41 (14.5) | 47 (16.6) | 16 (5.7) | 7 (2.5) |
| At 3 months | 283 (100) | 283 (100) | 229 (80.9) | 229 (80.9) | 39 (13.8) | 47 (16.6) | 15 (5.3) | 7 (2.5) |
| At 6 months | 226 (79.9) | 283 (100) | 57 (20.1) | 237 (83.7) | 0 | 42 (14.8) | 0 | 4 (1.4) |
| At 1 year | 199 (79.0) | 231 (88.5) | 55 (21.8) | 225 (86.2) | 0 | 19 (7.3) | 0 | 3 (1.1) |

DAPT, dual antiplatelet therapy; SAPT, single antiplatelet therapy

**Supplemental Figure 1.**

**
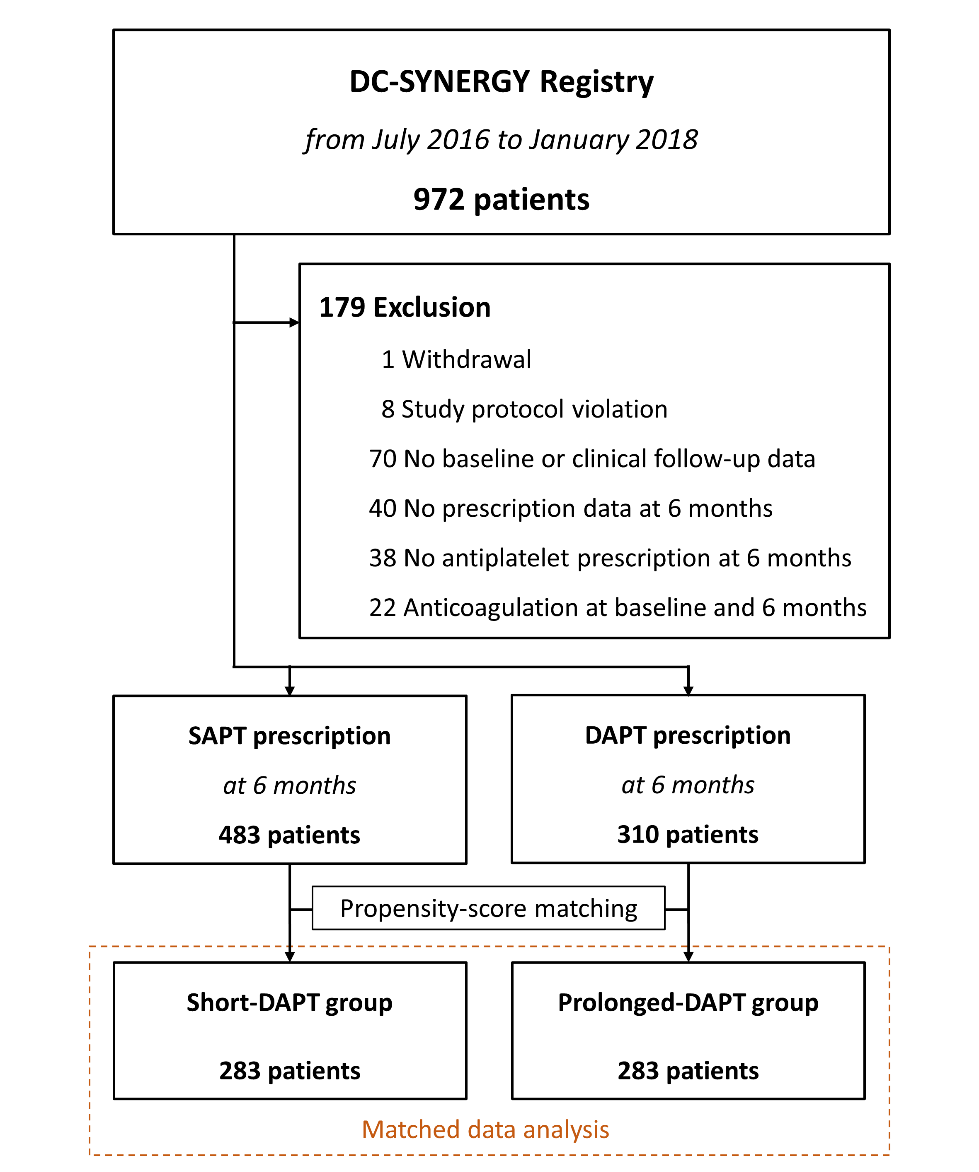
**

**Supplemental Figure 2.**


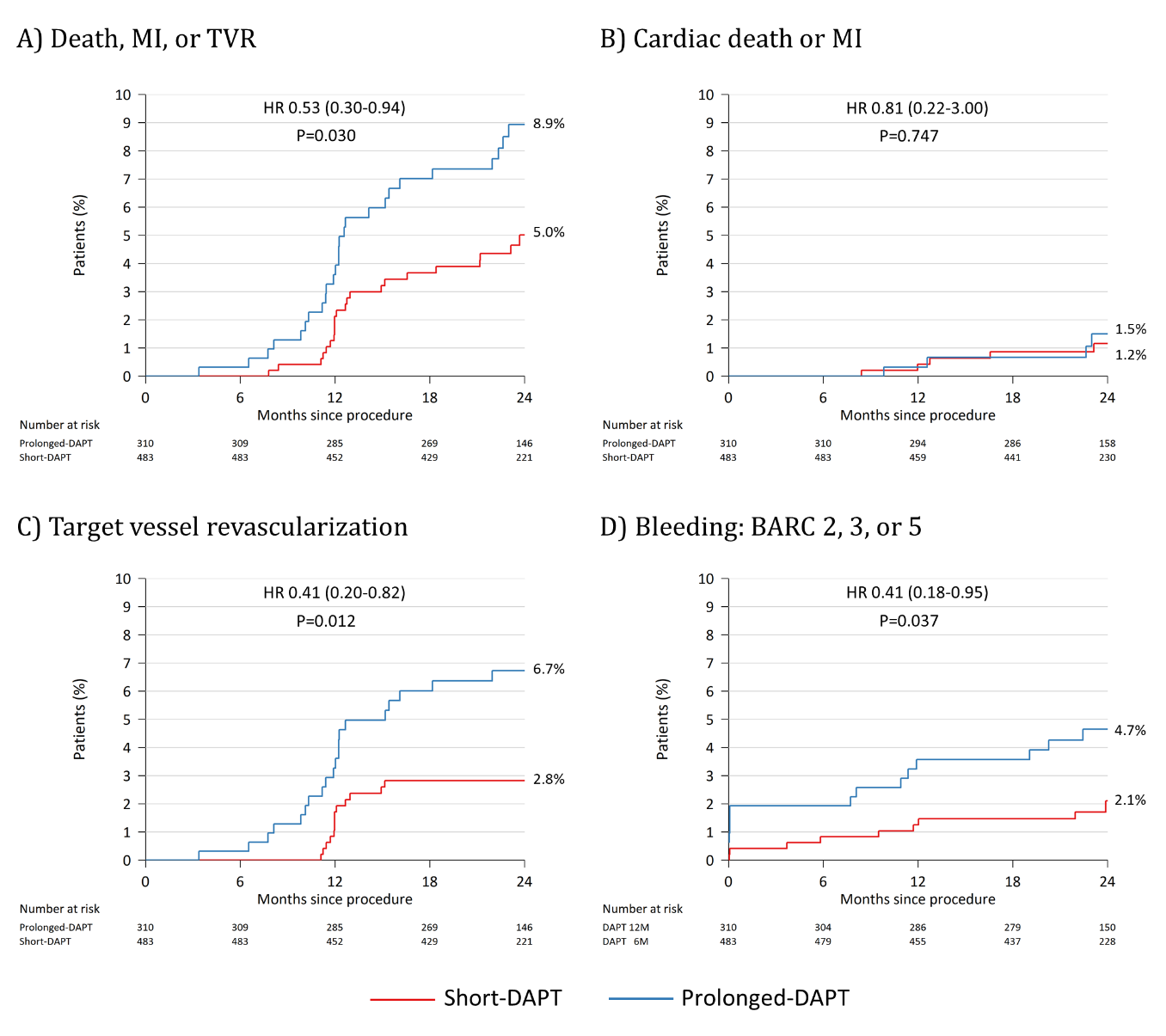

Supplement: Supplementary Materials — Supplemental Table 1: shows a higher rate of mortality, MI, or TVR in an unmatched population. Supplemental Table 2: shows the similar outcomes between short-DAPT and prolonged-DAPT groups after the inverse probability of treatment weighting. Supplemental Table 3: summarized the treads of antiplatelet uses in the study population. Supplemental Figure 1: shows the study flow. Supplemental Figure 2: reveals the Kaplan–Meier curves of unmatched population. Supplemental Table 1: primary and secondary endpoints between the short-DAPT and prolonged-DAPT groups in the total population. Supplemental Table 2: primary and secondary endpoints between the short-DAPT and prolonged-DAPT groups in the total population with inverse probability of treatment weighting. Supplemental Table 3: antiplatelet agents used during follow-up in the matched population. Supplemental Figure 1: Study Flow. Supplemental Figure 2: Kaplan–Meier survival curves for primary and secondary endpoints in the total population. [file 2914385.f1.doc]
